# 8-Cl-cAMP antagonizes mitogen-activated protein kinase activation and cell growth stimulation induced by epidermal growth factor

**DOI:** 10.1038/sj.bjc.6690820

**Published:** 1999-12

**Authors:** A Budillon, E Di Gennaro, M Caraglia, D Barbarulo, A Abbruzzese, P Tagliaferri

**Affiliations:** Istituto Nazionale dei Tumori, Fondazione ‘G Pascale’, Via M Semmola 80131, Napoli, Italy; Dipartimento di Biochimica e Biofisica, II Università di Napoli, Napoli, Italy; Dipartimento di Endocrinologia ed Oncologia Molecolare e Clinica, Facoltà di Medicina e Chirurgia, Università degli Studi Federico II, Napoli, Italy

**Keywords:** cAMP analogue, protein kinase A, EGF, MAPK

## Abstract

The growth factor-activated mitogenic pathways are often disregulated in tumour cells and, therefore, they can provide specific molecular targets for novel anti-tumour approaches. 8-Chloro-cAMP (8-Cl-cAMP), a synthetic cAMP analogue, is a novel anti-tumour agent that has recently undergone clinical evaluation. We investigated the effects of 8-CI-cAMP on the epidermal growth factor (EGF)/EGF receptor (EGF-R) signalling in human epidermoid cancer KB cells, which are responsive to the mitogenic stimulus of EGF. We found that the growth-promoting activity of EGF was completely abolished when EGF treatment was performed in combination with 8-CI-cAMP. The inhibition of the EGF-induced proliferation by 8-CI-cAMP was paralleled by the blockade of the EGF-stimulated activation of mitogen-activated protein kinases (MAPK), ERK-1 and ERK-2. Conversely, we found an increase of EGF-R expression and EGF-R tyrosine phosphorylation when KB cells were growth inhibited by 8-Cl-cAMP. Moreover, the activity of Raf-1 and MEK-1 protein kinases, the activators upstream MAPK in the phosphorylation cascade induced by EGF, was not modified in 8-Cl-cAMP-treated cells. We concluded that the impairment of KB cell response to EGF, induced by 8-Cl-cAMP, resides in the specific inhibition of MAPK/ERKs activity while the function of the upstream elements in the EGF-R signalling is preserved. © 1999 Cancer Research Campaign


British Journal of Cancer (1999) 81(7), 1134–1141
© 1999 Cancer Research Campaign
Article no. bjoc.1999.0820

### 8-Cl-cAMP antagonizes mitogen-activated protein kinase activation and cell growth stimulation induced by epidermal growth factor

**A Budillon[1], E Di Gennaro[1], M Caraglia[2], D Barbarulo[1], A Abbruzzese[2] and P Tagliaferri[3*]**

[1]Istituto Nazionale dei Tumori, Fondazione 'G Pascale', Via M Semmola 80131, Napoli, Italy; [2]Dipartimento di Biochimica e Biofisica, II Università di Napoli, Napoli, Italy; [3]Dipartimento di Endocrinologia ed Oncologia Molecolare e Clinica, Facoltà di Medicina e Chirurgia, Università degli Studi Federico II, Napoli, Italy

**Summary** The growth factor-activated mitogenic pathways are often disregulated in tumour cells and, therefore, they can provide specific molecular targets for novel anti-tumour approaches. 8-Chloro-cAMP (8-Cl-cAMP), a synthetic cAMP analogue, is a novel anti-tumour agent that has recently undergone clinical evaluation. We investigated the effects of 8-CI-cAMP on the epidermal growth factor (EGF)/EGF receptor (EGF-R) signalling in human epidermoid cancer KB cells, which are responsive to the mitogenic stimulus of EGF. We found that the growth-promoting activity of EGF was completely abolished when EGF treatment was performed in combination with 8-CI-cAMP. The inhibition of the EGF-induced proliferation by 8-CI-cAMP was paralleled by the blockade of the EGF-stimulated activation of mitogen-activated protein kinases (MAPK), ERK-1 and ERK-2. Conversely, we found an increase of EGF-R expression and EGF-R tyrosine phosphorylation when KB cells were growth inhibited by 8-Cl-cAMP. Moreover, the activity of Raf-1 and MEK-1 protein kinases, the activators upstream MAPK in the phosphorylation cascade induced by EGF, was not modified in 8-Cl-cAMP-treated cells. We concluded that the impairment of KB cell response to EGF, induced by 8-Cl-cAMP, resides in the specific inhibition of MAPK/ERKs activity while the function of the upstream elements in the EGF-R signalling is preserved. © 1999 Cancer Research Campaign

**Keywords:** cAMP analogue; protein kinase A; EGF; MAPK

Molecular alterations of the signal transduction pathway components are frequently associated with the transformation process and, therefore, they provide specific targets for novel antitumour approaches. In this regard, a widely studied pathway is the Ras-dependent mitogen activated protein kinases (MAPKs) cascade that transduces signals from the membrane growth factor-receptor tyrosine kinases to the nucleus where it triggers cell proliferation (see also Figure 6). The binding of epidermal growth factor (EGF) to its receptor induces the autophosphorylation of the receptor's tyrosine residues (Ullrich and Schlessinger, 1990) that recruit multiple cellular components such as the Grb2 adapter protein and the GTP/GDP exchange factor SOS (Denhardt, 1996). Such events result in the formation of a specific protein complex leading to the activation of $p21^{ras}$ GTPase (Denhardt, 1996). The GTP-bound form of $p21^{ras}$ induces the translocation to the cell membrane and the activation of the serine-threonine protein kinase Raf-1 (Marshall, 1996), which in turn activates a family of dual specificity kinases known as mitogen-activated/extracellular-signal regulated kinase kinase or MEK. MEK is the component of the cascade of phosphorylation events downstream of growth factor receptors, which finally stimulates the extracellular-signal-regulated kinases (ERKs) (Crews and Erikson, 1993). These enzymes, ERK-1 and ERK-2, belong to a subgroup of MAPKs, a serine/threonine kinases family, which require phosphorylation on both a threonine and a tyrosine residue for their activation and which transmit the mitogenic signal into the nucleus by regulating gene expression through the phosphorylation of a variety of transcription factors (Marshall, 1996). Several attempts have been made to inhibit this pathway whose components are individually capable of inducing malignant transformation when aberrantly controlled (Huang, 1997).

The interference on the Ras-dependent ERKs cascade by agents targeted on different signalling pathways is considered an additional therapeutical approach (Wu et al, 1993). In fact, several studies have reported that cyclic AMP (cAMP) elevating agents, which activate cAMP-dependent protein kinase (PKA), also induce phosphorylation of Raf-1, preventing its activation by $p21^{ras}$ and finally lead to the inhibition of the ERKs cascade (Cook and McCormick, 1993; Wu et al, 1993; Hordijk et al, 1994). However, it has also been reported that PKA-mediated inhibition of cell growth may occur through modulation of intracellular targets distinct from the ERKs cascade (McKenzie and Pouysségur, 1996). On the other hand, PKA is able to stimulate the ERKs cascade in PC12 and in epithelial cells (Frodin et al, 1994; Vossler et al, 1997), and it is also involved in non-ERKs-stimulated mitogenic pathways (Withers et al, 1995). Taken together, such findings suggest that cAMP and PKA may produce different and apparently conflicting effects on mitogen-activated signal transduction pathways.

8-Chloro-cAMP (8-Cl-cAMP), a synthetic cAMP analogue, is a powerful inhibitor of tumour cell growth both in vivo and in vitro (Tagliaferri et al, 1988; Cho-Chung 1989; Bosanquet et al, 1997;

*Received 17 May 1999*
*Revised 27 May 1999*
*Accepted 2 June 1999*

*Correspondence to:* A Budillon

*Present address: Dipartimento di Medicina Sperimentale e Clinica, Università 'Magna Grecia', Catanzaro, Italy

Fassina et al, 1997; Tortora et al, 1997*b*; Langdon et al, 1998). Moreover, phase I clinical studies on 8-Cl-cAMP have been recently completed in cancer patients (Tortora et al, 1995; Saunders et al, 1997). The molecular mechanism of the growth inhibitory action of 8-Cl-cAMP has been proposed to result from the selective modulation of the two isoenzymes of PKA, type I (PKA-I) and type II (PKA-II) (Cho-Chung et al, 1995). These enzymes appear to differently modulate cell growth and differentiation. PKA-I is overexpressed in tumour cells and is increased in normal cells upon exposure to mitogenic stimuli (Miller et al, 1993; Tortora et al, 1993; Cho-Chung et al, 1995), whereas PKA-II expression is typical of terminally differentiated tissues and growth-arrested cells (Schwartz and Rubin, 1985; Budillon et al, 1995; Cho-Chung et al, 1995). 8-Cl-cAMP treatment of tumour cells reduces PKA-I activity and concomitantly increases PKA-II activity (Rohlff et al, 1993; Budillon et al, 1995; Cho-Chung et al, 1995; Scala et al, 1995). Therefore, the catalytic activity of PKA may be affected by 8-Cl-cAMP through regulation of the expression of the two isoenzymes rather than by direct activation, as previously demonstrated (Scala et al, 1995). The wide range of cancer cells whose growth is inhibited by 8-Cl-cAMP suggests a common mechanism of action that is likely to involve perturbation of an ubiquitous signal transduction pathway that initiates proliferation in a broad number of different cell systems, such as the mitogen-activated Ras-dependent ERKs cascade. Moreover, some reports show that 8-Cl-cAMP may interfere with growth factor autocrine pathways and growth factor-induced transformation (Ciardiello et al, 1996; Bianco et al, 1997). Therefore, 8-Cl-cAMP represents a suitable tool for studying pharmacological interference on disregulated mitogenic pathways in tumour cells.

In the present study we have investigated the effects exerted by 8-Cl-cAMP on the EGF-mediated signal transduction pathway. We have selected as experimental model the KB epidermoid carcinoma cell line that is responsive to the mitogenic stimulus induced by EGF (Aboud-Pirak et al, 1988; Caraglia et al, 1995) and is growth inhibited by 8-Cl-cAMP treatment. We have found that the growth-promoting activity of EGF on KB cells was completely abolished by 8-Cl-cAMP. Furthermore, we have shown that this effect was associated to the specific inhibition of ERKs (ERK-1 and ERK-2) activity induced by 8-Cl-cAMP, while the function of the upstream components of the EGF-induced mitogenic pathway was preserved.

### MATERIALS AND METHODS

### Materials

8-Cl-cAMP (8-Chloro-cyclic adenosine 3′,5′-monophosphate, sodium salt) was provided by KP Flora, National Cancer Institute (NCI), National Institutes of Health (Bethesda, MD, USA) 8-Bromo-cAMP and 8-Chloro-adenosine (8-Cl-adenosine) were purchased from BioLog Life Science Institute (Bremen, Germany). [*methyl*-$^3$H]thymidine (25 Ci mmol$^{-1}$), [$^{125}$I]EGF (30 µCi mg$^{-1}$) and [γ$^{32}$P]ATP (3000 Ci mmol$^{-1}$) were from Amersham (Buckinghamshire, UK). Protease inhibitors, receptor grade EGF, PMA, and forskolin were from Sigma Chemical Co. (St Louis, MO, USA). The anti-phosphotyrosine 4G10 and anti-PLC-γ1 mouse monoclonal antibodies (mAbs) were purchased from Upstate Biotechnology Inc. (Lake Placid, NJ, USA). The antisera anti-Raf-1, anti-ERK-1, anti-ERK-2, anti-MEK-1, anti-EGF-R and the anti-EGF-R 528 mAb were purchased from Santa Cruz Biotechnology Inc. (Santa Cruz, CA, USA). Anti-CDK4 polyclonal antibody is from Pharmigen (Missisauga, Canada). Rabbit and mouse Ig, horseradish peroxidase-linked (HRP) antibodies and enhanced chemiluminescence (ECL) immunodetection reagents were from Amersham (Buckinghamshire, UK). Protein-A-Sepharose CL-4B is from Pharmacia (Uppsala, Sweden). All media, serum, antibiotics and glutamine are from Gibco (Grand Island, NY, USA).

### Cell culture and cell proliferation assays

The human epidermoid carcinoma KB cell line, obtained from American Type Tissue Culture Collection (Rockville, MD, USA), was grown in Dulbecco's modified Eagle's medium (DMEM) supplemented with 10% heat inactivated fetal bovine serum, penicillin (50 units ml$^{-1}$), streptomycin (500 µg ml$^{-1}$), 20 mM HEPES, pH 7.4, and 4 mM glutamine, in a humidified atmosphere of 95% air and 5% carbon dioxide at 37°C. KB cells throughout all the experiments have been maintained in serum-containing medium and no EGF deprivation has been performed to enhance EGF effects on cell growth or protein phosphorylation. For cell growth assays $1.5 \times 10^5$ cells were seeded in triplicate in 6-multiwell (Corning Glass, Corning, NY, USA), 8-Cl-cAMP and/or EGF was added at indicated concentrations after 3 h from seeding. At selected times cell growth assessment was performed by haemocytometric cell count and Trypan blue viability assay, following gentle trypsinization. The statistical significance of the difference in cell growth with and without the addition of 8-Cl-cAMP and EGF was evaluated by analysis of variance. Statistical evaluation was performed with the BMDP statistical software package.

### Determination of DNA synthesis

A total of $2 \times 10^4$ cells well$^{-1}$ were incubated in 24-multiwell for 48 h with or without 8-Cl-cAMP, in the presence or absence of the indicated concentrations of EGF for the last 18 h, and then were pulsed with 0.5 µCi [*methyl*-$^3$H]thymidine (25 Ci mmol$^{-1}$) for 5 h. Incubations were stopped by washing cells four times with phosphate-buffered saline (PBS) followed by fixation with 5% ice-cold trichloroacetic acid (TCA) at 4°C for 30 min. Cells were then washed twice with ethanol and dissolved in 20 mM sodium hydroxide and 1% sodium dodecyl sulphate (SDS) and finally transferred to scintillation solution (Packard, Meriden, CT, USA) for radioactivity determination. The statistical significance of the difference in thymidine incorporation with and without 8-Cl-cAMP and EGF was evaluated by analysis of variance. Statistical evaluation was performed with the BMDP statistical software package.

### Tyrosine phosphorylation and immunoblotting

To characterize the pattern of tyrosine phosphorylation, KB cells were grown for 48 h with or without 10 µM 8-Cl-cAMP and were exposed for the indicated times to 10 nM EGF at 37°C. After harvesting by scraping and centrifugation, cells were washed once in PBS and lysed in buffer A (50 mM HEPES, 150 mM sodium chloride (NaCl), 1.5 mM magnesium chloride ($MgCl_2$), 5 mM EGTA, 1% glycerol, 1% Triton X-100, 20 mM sodium pyrophosphate, 10 mM sodium orthovanadate, 25 mM NaF, 1 mM phenylmethyl sulphonyl fluoride (PMSF), 0.4 mg ml$^{-1}$ aprotinin, 10 µg ml$^{-1}$ leupeptin). Equal volumes of sample buffer (62.5 mM

Tris–HCl pH 6.8, 20% glycerol, 2% SDS, 5% β-mercaptoethanol, 0.5% (w/v) bromophenol blue) were added to the samples and heated at 95°C for 3 min. Forty micrograms of total protein from each sample were separated by 10% SDS polyacrylamide gel electrophoresis (PAGE) and then transferred to nitrocellulose paper. The membranes were immunoblotted with anti-phosphotyrosine 4G10 mAb and probed with HRP-linked sheep anti-mouse IgG. Detection by ECL was performed as recommended in the manufacturer's instructions. For EGF-R autophosphorylation and PLC-γ1 tyrosine-phosphorylation, 0.5 mg of cell lysates were immunoprecipitated with anti-EGF-R 528 and anti-PLC-γ1 mAbs respectively and immune complexes were isolated by protein A and protein G sepharose respectively. Immunoprecipitated proteins were eluted by boiling the immune complexes in 25 μl sample buffer and resolved by 8% SDS-PAGE. Immunoblots with anti-phosphotyrosine mAb were prepared and probed as previously described. Primary and secondary antibodies were then extracted from membranes using buffer B (62.5 mM Tris–HCl pH 6.7, 100 mM β-mercaptoethanol and 2% SDS) and reprobed with anti-EGF-R polyclonal Ab or anti-PLCγ1 mAb, HRP anti-rabbit or anti-mouse IgG respectively and ECL. CDK4, ERK-1, ERK-2 and MEK-1 immunoblots were performed as described above, on total cell lysates subjected to 10% SDS-PAGE. Where indicated, 10% acrylamide gels containing 2% SDS were used, to improve resolution of closely migrating forms of ERK or MEK.

### Protein kinase assays

KB cells were cultured and treated as described above. MAPK activity assay was performed on immune-complexes as previously described (Kharbanda et al, 1994). In brief, cells were washed twice with ice-cold PBS, scraped and 1ysed for 1 h at 4°C in the buffer C (10 mM Tris, 150 mM NaCl, 2 mM EGTA, 2 mM dithiothreitol (DTT), 1 mM sodium orthovanadate, 1 mM PMSF, 10 μg $ml^{-1}$ aprotinin, 10 μg $ml^{-1}$ leupeptin and 1% glycerol) clarified by centrifugation and equivalent aliquots were incubated with anti-ERK-1 or anti-ERK-2 antisera and protein A-Sepharose for 16 h at 4°C. The immunoprecipitate was washed twice with buffer C and twice with buffer D: 20 mM HEPES pH 7.5, 10 mM magnesium acetate, 100 μM ATP, 1 mM sodium orthovanadate, 1 mM PMSF, 10 μ $ml^{-1}$ aprotinin, 10 μg $ml^{-1}$ leupeptin. The immunocomplex was then suspended in 30 μl buffer A containing 10 μCi of [$\gamma^{32}$P]ATP and 10 μg of myelin basic protein (MBP, Sigma) and the reaction allowed to proceed for 30 min at 30°C. An equal volume of 2 × sample buffer was then added and the samples were incubated at 90°C for 5 min. The proteins were separated by SDS-12.5% PAGE that was stained with Coomassie blue, dried and then subjected to autoradiography. Alternatively the reaction mixtures were stopped with the addition of 2.94% (w/v) orthophosphoric acid and red carmosin solution, centrifuged for 15 s and then spotted onto phosphocellulose filters (Whatman P81). Filters were washed 3 times in 1% acetic acid, air-dried and then counted by liquid scintillation using Omnifluor/toluene (DuPont-New England Nuclear, Boston, MA, USA). The statistical significance of the difference in ERK-1 or ERK-2 activity with and without 8-Cl-cAMP was evaluated by analysis of variance. Statistical evaluation was performed with the BMDP statistical software package.

Assay for Raf-1 kinase activity was performed by immunocomplex kinase assay as already described for MAPK using anti-Raf-1 antiserum. H1 histone (10 μg) (Upstate Biotechnology Inc.) was used as substrate, as previously described (Kharbanda et al, 1994) in 30 μl of buffer A containing 10 μCi of [$\gamma^{32}$P]ATP, the reaction was allowed to proceed for 30 min at 30°C and the proteins were separated by SDS-12.5% PAGE. To measure MEK-1 kinase activity cell lysates were prepared as described before by immunocomplex kinase assay using anti-MEK-1 antiserum. A substrate containing Thr-183 and Tyr-185 within the regulatory sequence common to ERK-1 and ERK-2 (Santa Cruz Biotechnology Inc, Santa Cruz, CA, USA) was used and the proteins were separated by SDS-15% PAGE.

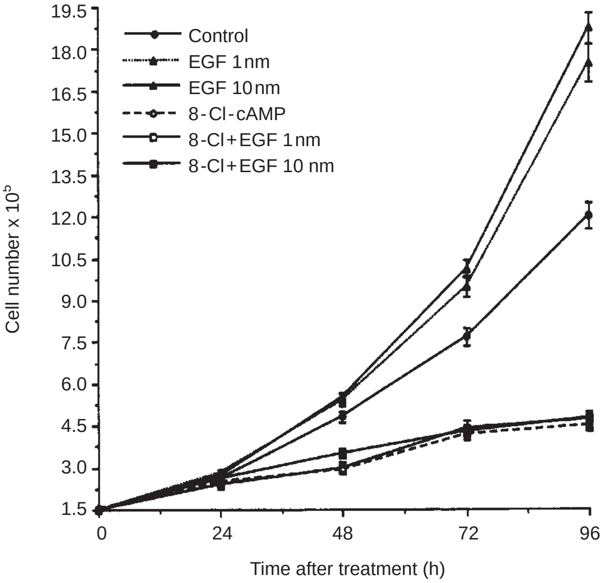

**Figure 1** Effects of 8-Cl-cAMP on cell growth stimulation induced by EGF in KB cells. Cells were seeded at $1.5 \times 10^5$ cells $well^{-1}$ in 6-well culture plates and exposed for different time points to two concentrations of EGF (1 and 10 nM) in the absence or presence of 10 μM 8-Cl-cAMP. Cell growth assessment was performed by haemocytometric cell counts following gentle trypsinization. The data show means of three separate experiments of duplicate determinations. The difference between the values of 8-Cl-cAMP or EGF-treated cells versus untreated cells were statistically significant ($P < 0.005$). Cell viability, assessed by Trypan blue, was consistently above 80%. Bars, s.d.

### RESULTS

### Effects of 8-Cl-cAMP on EGF-induced cell growth

In order to assess if 8-Cl-cAMP interferes with EGF mitogenic signalling in KB cells, we examined the effect of 8-Cl-cAMP on the EGF-induced tumour cell proliferation. A cell proliferation assay, using two doses of EGF in the presence or absence of 10 μM 8-Cl-cAMP for different times, is presented in Figure 1. EGF alone, at concentrations between 1 nM and 10 nM, which is the concentration range near the $K_d$ values of the low affinity receptors, brought about the maximal stimulation of KB cell growth after 96 h. However, the proliferative response to EGF was completely abolished in the presence of 8-Cl-cAMP. 8-Cl-cAMP alone induced up to 70% growth inhibition after 96 h (Figure 1), which occurred without citotoxicity as demonstrated by Trypan blue assay (data not shown). Since KB cells can be growth-stimulated by EGF even in the presence of serum (Aboud-Pirak et al, 1988) and in order to mimic more physiological conditions, cells were allowed to grow in 10% serum throughout this and all further

 *© 1999 Cancer Research Campaign*

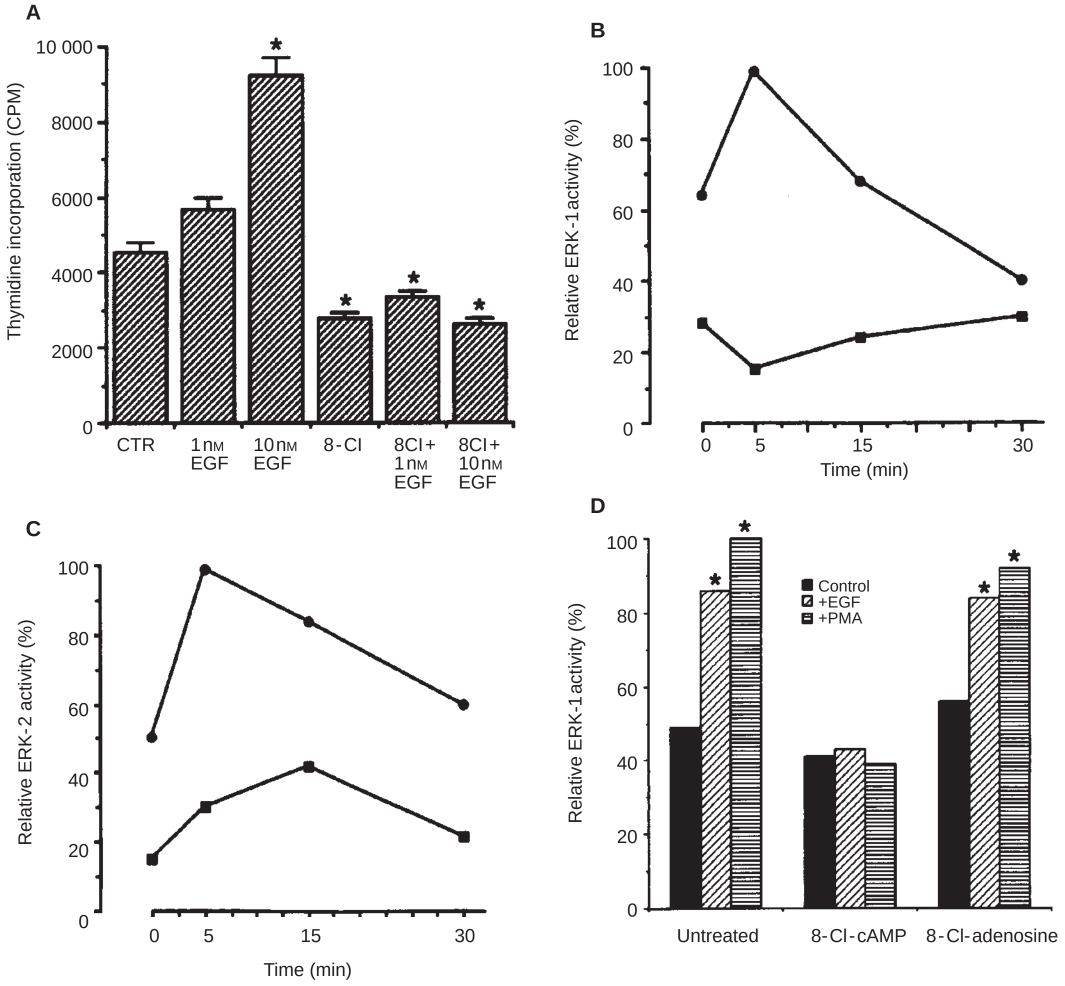

**Figure 2** Effects of 8-Cl-cAMP on DNA synthesis and MAPK activation induced by EGF. (**A**) KB cells growing in exponential phase in the presence or absence of 10 μM 8-Cl-cAMP for 48 h were stimulated with different concentrations of EGF for the last 18 h and [$^3$H]thymidine was added for the last 6 h. Incorporation of thymidine was detected on harvested cells by a liquid scintillation counter. Values are the means of three separate experiments of triplicate determinations; Bars, s.d.; *$P < 0.005$ as compared to untreated cells (CTR). (**B**) ERK-1 activity, (**C**) ERK-2 activity. Cells have been cultured for 48 h in the absence (●) or presence of 10 μM 8-Cl-cAMP (■) and exposed for the indicated times to 10 nM EGF. ERK-1 or ERK-2 activity is shown as per cent of the maximum activity (the value at 5 min of EGF treatment) and was determined by immunocomplex kinase assay as described in Materials and Methods using MBP as substrate. Each data point is the average of three separate experiments of triplicate determinations; standard errors never exceeded 10%; for each time point the difference between 8-Cl-cAMP-treated and untreated cells was statistically significant ($P < 0.005$, except for 30 min time point in **B**). (**D**) PMA- and EGF-induced ERK-1 activity in 8-Cl-cAMP- or 8-Cl-adenosine-treated KB cells. Cells cultured as described before, were untreated, treated with 10 μM 8-Cl-cAMP, or treated with 1 μM of 8-Cl-adenosine for 48 h and then stimulated for 5 min with 10 nM EGF or for 20 min with 100 nM PMA. ERK-1 activity was determined as described before and is expressed as percent of the maximum activity (the value at 20 min of PMA treatment). Results are representative of three independent experiments in triplicate determinations; standard errors never exceeded 10%; *$P<0.005$ as compared to controls experiments. Moreover, when cells were starved for 24 h and treated in serum-free medium, while the kinetic of EGF stimulation appeared different, probably because the interference of autocrine growth factor stimulation activated by the starvation stress, similar results were obtained for 8-Cl-cAMP effect (data not shown).

### Effect of 8-Cl-cAMP on DNA synthesis and ERKs activation

DNA synthesis and ERKs activation are early events in EGF growth-promoting activity (Marshall, 1996). DNA synthesis in response to EGF was measured by [$^3$H]thymidine incorporation assay. Maximal stimulation of [$^3$H]thymidine incorporation was obtained after exposure to 10 nM EGF for 18 h. When KB cells were incubated with 8-Cl-cAMP (10 μM) for 48 h in the presence of EGF for the last 18 h, the EGF-induced DNA synthesis was completely blocked (Figure 2A).

ERK-1 and ERK-2 activities were measured by an immunocomplex-kinase assay using the myelin basic protein (MBP) as substrate. As shown in Figure 2B and 2C, maximal activation of ERK-1 and ERK-2 was achieved 5 min after the addition of EGF while a specific inhibition of basal and EGF-induced activity was demonstrated in 8-Cl-cAMP-treated cells. ERK-1 stimulation by EGF was completely abrogated by 8-Cl-cAMP while ERK-2 activation was significantly reduced (Figure 2 B, C).

In order to study whether 8-Cl-cAMP inhibited specifically EGF-induced ERK-1 and ERK-2 activity or could be replicated in cells stimulated also by other ERK activators, MAPK activity was evaluated after exposure of both control and 8-Cl-cAMP-treated cells to phorbol 12-myristate 13-acetate (PMA). As shown in Figure 2D, 8-Cl-cAMP completely inhibited the stimulation of

© 1999 Cancer Research Campaign 

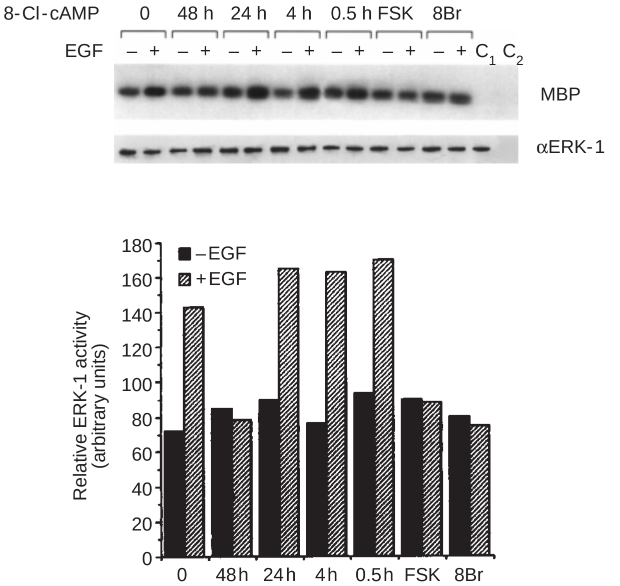

**Figure 3** Time course of 8-Cl-cAMP effect on EGF-induced ERK-1 activation. KB cells growing in exponential phase were untreated or treated with 10 μM 8-Cl-cAMP for the indicated times, with 25 μM forskolin or 0.5 mM 8-Br-cAMP for 15 min, and then stimulated for 5 min with 10 nM EGF. ERK-1 was immunoprecipitated and analysed for kinase activity using immunocomplex kinase assay and, for ERK-1 protein expression, by Western blotting, as described in Materials and Methods. The lower panel shows the ERK-1 relative activity calculated by laser scanner densitometry and normalized for protein levels. The experiment shown is one of three experiments that gave similar results

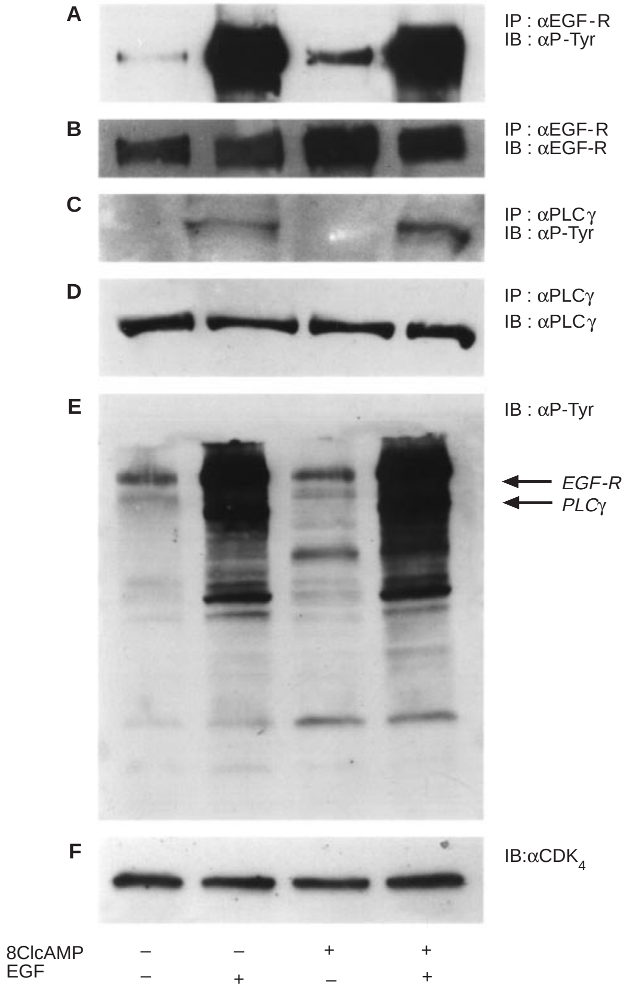

**Figure 4** EGF-induced tyrosine phosphorylation of EGF-R, PLCγ1 and whole cell proteins in 8-Cl-cAMP-treated cells. KB cells growing in exponential phase in the presence or absence of 10 μM 8-Cl-cAMP for 48 h, were stimulated with 10 μM EGF for 5 min. Immunoprecipitated complex performed with the indicated antibodies (IP), were electrophoresed on SDS-PAGE, as described in Materials and Methods, transferred to nitrocellulose membranes, immunostained with the indicated antibodies (IB) and detected by ECL. Each experiment shown is representative of at least three experiments that gave similar results

ERK-1 activity in response to both EGF and PMA. Moreover, it was previously shown that 8-Cl-cAMP can be hydrolysed to 8-Cl-adenosine by the serum-phosphodiesterases and 5′-nucleotidase and has been hypothesized that the latter molecule can be indeed responsible for the effects of 8-Cl-cAMP (Lange-Carter et al, 1993; Langeveld et al, 1997). However, in our experimental conditions, the inhibition of ERK-1 activity induced by 8-Cl-cAMP was not due to the metabolite because 8-Cl-adenosine, while inducing over 50% cell growth inhibition (data not shown), did not modify EGF-stimulated or PMA-stimulated ERK-1 activity (Figure 2D).

A time-course evaluation of the effects exerted by 8-Cl-cAMP on EGF-induced ERKs activation demonstrated that inhibition occurred only after 48 h treatment, whereas the treatment for 24 h, 4 h or 30 min had no effect (Figure 3) even using concentrations as high as 1 mM of 8-Cl-cAMP (data not shown). Conversely, treatment for 30 min with the cAMP elevating agent forskolin or with the cAMP analogue 8-Br-cAMP, both PKA activators, blocked ERKs activation (Figure 3), confirming previous results (Cook and McCormick, 1993; Wu et al, 1993; Hordijk et al, 1994). Anti-ERK-1 immunoblot was used for normalization of the immunocomplex kinase assay and the ERK-1 relative activity is shown in Figure 3. As a consequence of these results we carried out all further experiments exposing the cells to 8-Cl-cAMP for 48 h.

### Effects of 8-Cl-cAMP on EGF signalling

Since 8-Cl-cAMP antagonized the growth-promoting activity of EGF and inhibited the EGF-induced ERKs activation, we investigated the effects of this compound on the components of the EGF-induced signalling located upstream ERKs. We investigated whether the inhibition of EGF-induced ERKs activation and DNA synthesis, exerted by 8-Cl-cAMP, could be dependent on the decreased expression or function of EGF-R. We tested the effect of 8-Cl-cAMP on EGF-R activation by studying the EGF-induced receptor autophosphorylation (Figure 4A). Treatment of the cells with 10 nM EGF for 10 min induced a 20-fold increase of EGF-R tyrosine phosphorylation as determined by laser scanner densitometry of Western blot data. Surprisingly, basal EGF-R tyrosine phosphorylation in 8-Cl-cAMP-treated cells was threefold increased and, upon EGF treatment, it further increased up to 25-fold. This effect was paralleled by a threefold increase of EGF-R expression (Figure 4B). We also examined tyrosine phosphorylation of phospholipase C-γ1 (PLCγ1) which is catalysed by the activated EGF-R-tyrosine kinase (Denhardt, 1996). As shown in Figure 4C, EGF induced PLCγ1 tyrosine phosphorylation in control as well as in 8-Cl-cAMP-treated cells while immunoblot with anti-PLCγ1 antiserum revealed equal amount of PLCγ1 protein in all immunoprecipitates (Figure 4D). Finally, we measured the effect of 8-Cl-cAMP on EGF-induced tyrosine

 © 1999 Cancer Research Campaign

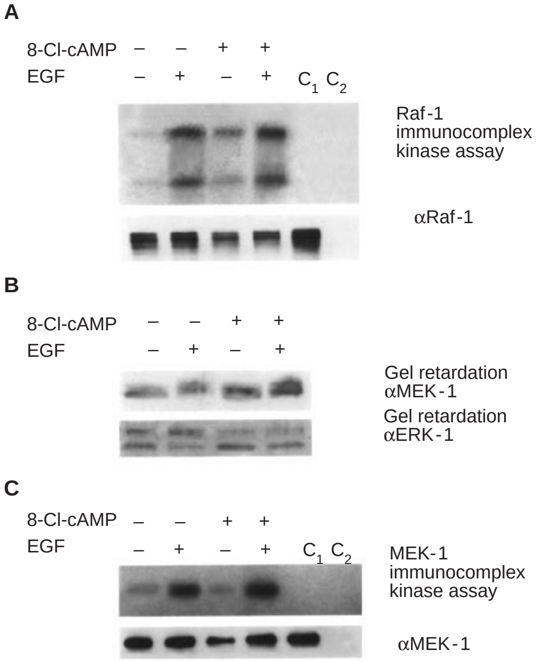

**Figure 5** Raf-1 and MEK-1 kinase activity in 8-Cl-cAMP-treated cells. KB cells growing in exponential phase in the presence or absence of 10 μM 8-Cl-cAMP for 48 h, were stimulated with 10 μM EGF for 5 min. (**A**) Raf-1 was immunoprecipitated and analysed for kinase activity by immunocomplex kinase assay using histone H1 as substrate (Kharbanda et al, 1994). For Raf-1 protein expression a Western blotting was performed, as described in Materials and Methods. Lane C1 indicated a control where the immunocomplex kinase assay was performed on untreated EGF-stimulated cells in the absence of the substrate. Lane C2 indicated a control assay performed using non-immune serum to immunoprecipitate untreated EGF-stimulated cells. (**B**) MEK-1 and ERK-1 activity was evaluated by gel retardation experiments using 10% acrylamide gels containing 2% SDS to improve resolution of closely migrating forms. (**C**) MEK-1 was immunoprecipitated and analysed for kinase activity by immunocomplex kinase assay using as substrate a peptide containing Thr-183 and Tyr-185 within the regulatory sequence common to ERK-1 and ERK-2 (Santa Cruz Biotechnology Inc, Santa Cruz, CA, USA). For MEK-1 protein expression a Western blotting was performed, as described in Materials and Methods. C1 and C2 control samples have been assayed as described for Raf-1. Each experiment shown is representative of at least three experiments that gave similar results.

phosphorylation of whole cell lysate proteins. As shown in Figure 4E, treatment with 8-Cl-cAMP alone increased basal tyrosine phosphorylation while the same extent of EGF-stimulated tyrosine phosphorylation was demonstrated in control as well as in 8-Cl-cAMP-treated cells. Cyclin-dependent kinase 4 (CDK4) protein expression was evaluated by Western blotting as control for equal protein loading (Figure 4F).

It was recently reported that the PKA-induced phosphorylation of Raf-1 kinase prevented the interaction with membrane-bound activated p21$^{ras}$ inducing the inhibition of the ERKs cascade (Wu et al, 1993). In order to study the effect of 8-Cl-cAMP on Raf-1 kinase activity we performed an immunocomplex kinase assay using H1 histone as Raf-1 substrate (Kharbanda et al, 1994). As shown in Figure 5A, treatment of KB cells for 48 h with 10 μM 8-Cl-cAMP did not affect EGF-induced Raf-1 kinase activity. The latter finding indicated again that the mechanism of 8-Cl-cAMP-induced inhibition of ERKs is different from the previously reported PKA-mediated mechanism of action (Wu et al, 1993).

In the EGF-induced pathway Raf-1 kinase activates MAPK/Extracellular signal-regulated kinase also known as MEK, which in turn induces activation of ERKs (Denhardt, 1996). Therefore, to further characterize the mechanism of ERKs inhibition by 8-Cl-cAMP, we evaluated EGF-induced MEK-1 activity in control as well as in 8-Cl-cAMP-treated cells. As shown in Figure 5B, treatment of KB cells for 48 h with 10 μM 8-Cl-cAMP did not affect EGF-induced phosphorylation of MEK-1 as detected on anti-MEK-1 immunoblot where the phosphorylated form of MEK showed a slower electrophoretic mobility on a 10% acrylamide gel containing 2% SDS (see 'Material and Methods'). However, after stripping and reprobing the same blot with anti-ERK-1 antiserum the EGF-stimulated active form of ERK-1, which showed reduced mobility, disappeared in 8-Cl-cAMP-treated cells (Figure 5B, lower panel). MEK-1 activity was also evaluated by an immunocomplex kinase assay using a specific peptide substrate containing Thr-183 and Tyr-185 within the regulatory sequence common to ERK-1 and ERK-2. As shown in Figure 5C, we could not detect any change in EGF-induced MEK-1 kinase activity or MEK-1 protein expression upon 8-Cl-cAMP treatment.

### DISCUSSION

In this work we have shown that 8-Cl-cAMP antagonizes the EGF-induced cell proliferation and DNA synthesis in KB epidermoid cancer cells by interfering with ERKs activation, which is a crucial event in the signalling cascade of the EGF-mediated mitogenic pathway (Denhardt, 1996).

We have challenged the hypothesis that the impairment of KB cell response to EGF could be due to a down-regulation of the EGF-R expression or function. However, EGF-R expression was not reduced by 8-Cl-cAMP that, conversely, induced an increase in the cellular content of EGF-R. Upregulation of EGF-R expression by 8-Cl-cAMP was paralleled by an increase in tyrosine phosphorylation of EGF-R and of total cell protein in treated cells. Therefore, 8-Cl-cAMP enhanced the activation of the early components in the EGF-induced signalling cascade while it neutralized the EGF-stimulated cell growth. Moreover, we have recently demonstrated that 8-Cl-cAMP induces an increase in EGF-R surface expression, which is dependent on an increase in EGF-R synthesis (Caraglia et al, 1999). We think that these apparently contradictory results could be, at least in part, explained by several previous findings from our group and others. In fact, we have reported that an upregulation of EGF-R is associated to the growth inhibition of human tumour cells induced by interferon-α (IFN-α) or by anticancer drugs, such as 1-β-D-arabinofuranosylcytosine (ARA-C), used at cytostatic concentrations (Budillon et al, 1991; Caraglia et al, 1993). EGF-R up-regulation has also been reported after tumour cell treatment with low doses of adriamycin or retinoic acid (Jetten, 1980; Zuckier and Tritton, 1983). Furthermore, IFN-α, but not ARA-C, potentiates the growth-promoting effect of EGF in KB cells while inducing EGF-R up-regulation (Caraglia et al, 1995). On this basis, we have hypothesized that up-regulation of growth factor receptors is a common event in growth-inhibited tumour cells and could represent a protective response towards the antiproliferative stimuli (Tagliaferri et al, 1994). This non-specific response could, however, result in sensitization to EGF only after treatment of tumour cells by physiological molecules such as IFN-α.

The key finding of the present study is that 8-Cl-cAMP while inducing EGF-R up-regulation, this effect being analogous to other growth inhibiting compounds, also inhibited EGF-stimulated ERKs activity and antagonized the mitogenic effect of EGF.

© 1999 Cancer Research Campaign

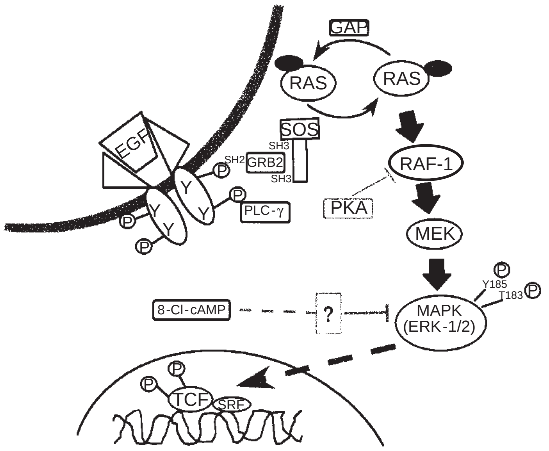

**Figure 6** A simplified scheme of the EGF-induced Ras-dependent ERKs cascade and the hypothetical site of 8-Cl-cAMP inhibitory effect. The MAPK isoforms ERK-1 and ERK-2 upon stimulation by the dual specificity kinase MEK, which phosphorylates on both threonine and tyrosine residues, transmit the mitogenic signal migrating into the nucleus and regulating gene expression through the phosphorylation of a variety of transcription factors (TCF) and the activation of serum response element (SRE). Analysis of the mitogen-regulating signals placed MAPK (ERK1/2) in an apparently linear signalling cascade downstream of growth factor receptors, adaptor proteins (Grb2), guanine nucleotide exchange factors (SOS), Ras, Raf-1 and MEK, most of which are capable of cellular transformation when aberrantly controlled. PKA can prevent Raf-1 activation by $p21^{ras}$ and can inhibit growth factor-induced ERKs activation. Our hypothesis is that 8-Cl-AMP has a different mechanism of action for ERKs cascade inhibition, since act downstream Raf and MEK. More details are in the text

Furthermore, the 8-Cl-cAMP inhibition of EGF-induced mitogenic signalling was not dependent on Raf-1 kinase inhibition, and occurred upstream ERKs but downstream MEK. In fact, we have found that neither activation nor expression of MEK, which lays downstream Raf-1 and is the direct activator of ERKs (Denhardt, 1996; Marshal, 1996), were affected by 8-Cl-cAMP treatment.

The mechanism by which 8-Cl-cAMP interfered with ERKs activation is not known. The results presented in this study suggest a selective inhibition of 8-Cl-cAMP on ERKs or on a still unknown upstream activator (Figure 6). While several reports have demonstrated the possibility of by-passing some steps of the cascade of events induced by EGF and ultimately activating ERKs (Faure and Bourne, 1995; Csar et al, 1997), to our knowledge no other specific activator except MEK has been shown to directly stimulate ERKs.

Our findings could be interpreted on the basis of the unique mechanism of action of 8-Cl-cAMP in down-regulating PKA-I isoenzyme. Since PKA-I expression has been related to cell proliferation and transformation (Miller et al, 1993; Tortora et al, 1993; Cho-Chung et al, 1995) and 8-Cl-cAMP down-regulates the expression of RIα subunit of PKA in KB cells (Budillon et al, unpublished observation), this effect could interfere with EGF-induced signalling. In this regard, Tortora et al reported that RIα subunit binds to Gbr2 and allows the interaction of PKA-I with the activated EGF-R in human breast MCF-10 cells (Tortora et al, 1997*a*), suggesting a possible interference of PKA-I inhibitors with the early steps of EGF-R-mediated signalling. In our system we did not examine if this interaction is present or if it is regulated by 8-Cl-cAMP. However, we demonstrated that this agent had no effect on the early steps of EGF-R-mediated signalling. In fact, our data suggest that, even if the 8-Cl-cAMP effect on ERKs activation is mediated through RIα down-regulation, this effect does not occur by depressing the function or the cellular content of EGF-R in our epidermoid tumour cell system.

Furthermore, it has been hypothesized that 8-Cl-adenosine, the metabolite derived from 8-Cl-cAMP phosphodiesterase-mediated degradation, can be responsible for the growth-inhibitory effects of this agent (Lange-Carter et al, 1993; Langeveld et al, 1997). However, other investigations have demonstrated that the anti-tumour activity of 8-Cl-cAMP is not dependent on its metabolite (Tagliaferri et al, 1988; Rohlff et al, 1993; Cho-Chung et al, 1995; Noguchi et al, 1998). Moreover, in our experimental conditions, the effects of 8-Cl-cAMP were not mediated by its metabolite, since 8-Cl-adenosine did not modify EGF-stimulated or PMA-stimulated ERK-1 activity.

Several examples of a cross-talk between the transduction pathways induced by mitogenic stimuli and the PKA-activated signalling have been described (Cook and McCormick, 1993; Wu et al, 1993; Frodin et al, 1994; Hordijk et al, 1994; Vossler et al, 1997). It is well known that cAMP influences negatively the transmission of growth signal through the Ras pathway (Cook and McCormick, 1993; Wu et al, 1993; Hordijk et al, 1994). In particular, it has been suggested that PKA induced phosphorylation of Raf-1 reducing its ability to bind Ras and then blocking the ERKs signalling (Wu et al, 1993). However, 8-Cl-cAMP is not considered an activator of PKA, conversely it is rather an antagonist of PKA-I (Rohlff et al, 1993; Cho-Chung et al, 1995; Scala et al, 1995) which is the major isoenzyme expressed in KB cells (Budillon et al, unpublished observation). In addition, we have demonstrated that 8-Cl-cAMP had no effect on Raf-1 kinase activity in these cells.

Ciardiello et al (1996) have demonstrated a cooperative anti-tumour effect of 8-Cl-cAMP and anti-EGF-R MAb 528 in vivo on tumour xenografts. It was proposed that the cooperative effect is the result of a multiple step inhibition of the EGF-mediated signalling pathway induced by the two agents. If we consider that mAb 528 has receptor blocking activity it is conceivable that it could be potentiated by 8-Cl-cAMP which also hinder the EGF mitogenic pathway by selective ERKs inhibition. Furthermore, the up-regulation of EGF-R induced by 8-Cl-cAMP could enhance mab 528 binding to tumour cells.

In conclusion, our results add new insights into the mechanism of anti-tumour action of 8-Cl-cAMP demonstrating that this agent can specifically inhibit the terminal enzymes of EGF/EGF-R pathway in human cancer cells. Since 8-Cl-cAMP is currently being investigated as an anticancer agent in phase I studies (Tortora et al, 1995; Saunders et al, 1997), the identification of the specific molecular targets of 8-Cl-cAMP may allow the design of selective approaches in order to enhance the anti-tumour activity of this compound.

### ACKNOWLEDGEMENTS

This paper is dedicated to the memory of Dr Ciro Manzo. This study was partially supported by grants from AIRC (Associazione Italiana per la Ricerca sul Cancro). We are grateful to Dr Paolo Di Fiore, Dr Giuseppe Viglietto and Dr Ugo D'Oro for discussions and critical reading of the manuscript.

### REFERENCES

Aboud-Pirak E, Hurwitz E, Pirak ME, Bellot F, Schlessinger J and Sela M (1988) Efficacy of antibodies to epidermal growth factor receptor against KB carcinoma in vitro and in nude mice. *J Natl Cancer Inst* **80**: 1605–1611

 *© 1999 Cancer Research Campaign*

Bianco C, Tortora G, Baldassarre G, Caputo R, Fontanini G, Chinè S, Bianco AR and Ciardiello F (1997) 8-Chloro-cyclic AMP inhibits autocrine and angiogenic growth factor production in human colorectal and breast cancer. *Clin Cancer Res* **3**: 439–448
Bosanquet AG, Buriton AR, Bell PB and Harris AL (1997) Ex vivo cytotoxic drug evalutation by DISC assay to expedite identification of clinical targets: results with 8-chloro-cAMP. *Br J Cancer* **76**: 511–518
Budillon A, Tagliaferri P, Caraglia M, Torrisi MR, Normanno N, Iacobelli S, Palmieri G, Stoppelli MP, Frati L and Bianco AR (1991) Upregulation of epidermal growth factor receptor induced by alpha-interferon in human epidermoid cancer cells. *Cancer Res* **51**: 1294–1299
Budillon A, Cereseto A, Kondrashin A, Nesterova M, Merlo G, Clair T and Cho-Chung YS (1995) Point mutation of the autophosphorylation site or in the nuclear location signal causes protein kinase A RIIβ regulatory subunit to lose its ability to revert transformed fibroblasts. *Proc Natl Acad Sci USA* **92**: 10634–10638
Caraglia M, Tagliaferri P, Correale P, Genua G, Pinto A, Esposito G and Bianco AR (1993) Cytosine arabinoside increases the binding of [$^{125}$I]-labelled epidermal growth factor and [$^{125}$I]-transferrin and enhances the in vitro targeting of human tumour cells with anti-(growth factor receptor) mAb. *Cancer Immunol Immunother* **37**: 150–156
Caraglia M, Leardi A, Ciardiello F, Budillon A, Guarrasi R, Bianco AR and Tagliaferri P (1995) α-Interferon potentiates epidermal growth factor receptor-mediated effects on human epidermoid carcinoma KB cells. *Int J Cancer* **61**: 342–347
Caraglia M, Di Gennaro E, Barbarulo D, Marra M, Tagliaferri P, Abbruzzese A and Budillon A (1999) Upregulated EGF receptors undergo to rapid internalization and ubiquitin-dependent degradation in human cancer cells exposed to 8-Cl-cAMP. *FEBS Lett* **447**: 203
Cho-Chung YS (1989) Site-selective 8-chloro-cyclic adenosine 3′,5′-monophosphate as a biologic modulator of cancer: restoration of normal control mechanisms. *J Natl Cancer Inst* **81**: 982–987
Cho-Chung YS, Pepe S, Clair T, Budillon A and Nesterova M (1995) cAMP-dependent protein kinase: role in normal and malignant growth. *Crit Rev Oncol Hematol* **21**: 33–61
Ciardiello F, Damiano V, Bianco R, Bianco C, Fontanini G, De Laurentis M, De Placido S, Mendelsohn J, Bianco AR and Tortora G (1996) Antitumor activity of combined blockade of epidermal growth factor receptor and protein kinase A. *J Natl Cancer Inst* **88**: 1770–1776
Cook SJ and McCormick F (1993) Inhibition by cAMP of Ras-dependent activation of Raf. *Science* **262**: 1069–1072
Crews CM and Erikson RL (1993) Extracellular signals and reversible protein phosphorylation: what to MEK of it all. *Cell* **74**: 215–217
Csar XF, Ward AC, Hoffmann BW, Guy GG and Hamilton JA (1997) cAMP suppresses p21$^{ras}$ and Raf-1 responses but not the Erk-1 response to granulocyte-colony-stimulating factor: possible Raf-1-independent activation of Erk-1. *Biochem J* **322**: 79–87
Denhardt DT (1996) Signal-transduction protein phosphorylation cascades mediated by ras/rho proteins in the mammalian cell: the potential for multiplex signalling. *Biochem J* **318**: 729–747
Faure M and Bourne HR (1995) Differential effects of cAMP on the MAP kinase cascade: evidence for a cAMP-insensitive step that can bypass Raf-1. *Mol Biol Cell* **6**: 1025–1035
Fassina G, Aluigi MG, Gentleman S, Wong P, Cai T, Albini A and Noonan DM (1997) The cAMP analog 8-Cl-cAMP inhibits growth and induces differentiation and apoptosis in retinoblastoma cells. *Int J Cancer* **72**: 1088–1094
Frodin M, Peraldi P and Van Obberghen E (1994) Cyclic AMP activates the mitogen-activated protein kinase cascade in PC12 cells. *J Biol Chem* **269**: 6207–6214
Hordijk PL, Verlaan I, Jalink K and Moolenaar WH (1994) cAMP abrogates the p21ras-mitogen-activated protein kinase pathway in fibroblasts. *J Biol Chem* **269**: 3534–3538
Huang PS and Heimbrook DC (1997) Oncogene products as therapeutic targets for cancer. *Curr Opin Oncol* **9**: 94–100
Jetten AM (1980) Retinoids specifically enhance the number of epidermal growth factor receptors. *Nature (Lond)* **284**: 626–629
Kharbanda S, Saleem A, Emoto Y, Stone R, Rapp U and Kufe D (1994) Activation of Raf-1 and mitogen-activated protein kinases during monocytic differentiation of human myeloid leukemia cells. *J Biol Chem* **269**: 872–878
Lange-Carter CA, Vuillequez JJ and Malkinson AM (1993) 8-Chloro-adenosine mediates 8-chloro-cyclic AMP-induced down-regulation of cyclic AMP-dependent protein kinase in normal and neoplastic mouse lung epithelial cells by a cyclic AMP-independent mechanism. *Cancer Res* **53**: 393–400
Langeveld CH, Jongenelen CA, Theeuwes JW, Baak JP, Heimans JJ, Stoof JC and Peters GJ (1997) The antiproliferative effect of 8-chloro-adenosine, an active metabolite of 8-chloro-cyclic adenosine monophosphate, and disturbances in nucleic acid synthesis and cell cycle kinetics. *Biochem Pharmacol* **53**: 141–148
Langdon SP, Ritchie AA, Muir M, Dodds M, Howie AF, Leonard RC, Stockman PK and Miller WR (1998) Antitumour activity and schedule dependency of 8-chloro-adenosine- 3′,5′-monophosphate (8-Cl-cAMP) against human tumour xenografts. *Eur J Cancer* **34**: 384–388
Marshall CJ (1994) MAP kinase kinase kinase, MAP kinase kinase and MAP kinase. *Curr Opin Genet Dev* **4**: 82–89
Marshall CJ (1996) Raf gets it together. *Nature (Lond)* **383**: 127–128
McKenzie FR and Pouysségur J (1996) cAMP-mediated growth inhibition in fibroblasts is not mediated via mitogen-activated protein (MAP) kinase (ERK) inhibition. *J Biol Chem* **271**: 13476–13483
Miller WR, Hulme MJ, Cho-Chung YS and Elton RA (1993) Types of cyclic AMP binding proteins in human breast cancers. *Eur J Cancer* **29**: 989–991
Noguchi K, Murata T and Cho-Chung YS (1998) 8-chloroadenosine 3′,5′-monophosphate (8-Cl-cAMP) selectively eliminates protein kinase A type I to induce growth inhibition in c-ras-transformed fibroblasts. *Eur J Cancer* **34**: 1260–1267
Rohlff C, Clair T and Cho-Chung YS (1993) 8-Cl-cAMP induces truncation and down-regulation of the RI alpha subunit and up-regulation of the RII beta subunit of cAMP-dependent protein kinase leading to type II holoenzyme-dependent growth inhibition and differentiation of HL-60 leukemia cells. *J Biol Chem* **268**: 5774–5782
Saunders MP, Salisbury AJ, O'Byrne KJ, Long L, Whitehouse RM, Talbot DC, Mawer EB and Harris AL (1997) A novel cyclic adenosine monophosphate analog induces hypercalcemia via production of 1,25-dihydroxyvitamin D in patients with solid tumors. *J Clin Endocrinol Metab Dec* **82**: 4044–4048
Scala S, Budillon A, Zhan Z, Cho-Chung YS, Jefferson J, Tsokos M and Bates SE (1995) Downregulation of *mdr*-1 expression by 8-Cl-cAMP in multidrug resistant MCF-7 human breast cancer cells. *J Clin Inv* **96**: 1026–1034
Schwartz DA and Rubin CS (1985) Identification and differential expression of two forms of regulatory subunits (RII) of cAMP-dependent protein kinase II in Friend erythroleukemic cells Differentiation and 8-bromo-cAMP elicit a large and selective increase in the rate of biosynthesis of only one type of RII. *J Biol Chem* **260**: 6296–6303
Tagliaferri P, Caraglia M, Muraro R, Pinto A, Budillon A, Zagonel V and Bianco AR (1994) Pharmacological modulation of peptide growth factor receptor expression on tumor cells as a basis for cancer therapy. *Anticancer Drugs* **5**: 379–393
Tagliaferri P, Katsaros D, Clair T, Neckers L, Ally S, Tortora G, Neckers L, Rubalcava B, Parandoosh Z, Chang YA, Revankar GR and Cho-Chung YS (1988) Synergistic inhibition of growth of breast and colon human cancer cell lines by site-selective cyclic AMP analogues. *Cancer Res* **48**: 1642–1650
Tortora G, Ciardiello F, Pepe S, Tagliaferri P, Ruggiero A, Bianco C, Guarrasi R, Miki K and Bianco AR (1995) Phase I clinical study with 8-chloro-cAMP and evaluation of immunological effects in cancer patients. *Clin Cancer Res* **1**: 377–384
Tortora G, Damiano V, Bianco C, Baldassarre G, Bianco AR, Lanfrancone L, Pelicci PG, and Ciardiello F (1997*a*) The RIα subunit of protein kinase A (PKA) binds to Grb2 and allows PKA interaction with the activated EGF-receptor. *Oncogene* **14**: 923–928
Tortora G, Di Isernia G, Sandomenico C, Bianco R, Pomatico G, Pepe S, Bianco AR and Ciardiello F (1997*b*) Synergistic inhibition of growth and induction of apoptosis by 8-chloro-cAMP and paclitaxel or cisplatin in human cancer cell. *Cancer Res* **57**: 5107–5111
Tortora G, Pepe S, Cirafici AM, Ciardiello F, Porcellini A, Clair T, Colletta G, Cho-Chung YS and Bianco AR (1993) Thyroid-stimulating hormone-regulated growth and cell cycle distribution of thyroid cells involve type I isozyme of cyclic AMP-dependent protein kinase. *Cell Growth Differ* **4**: 359–365
Ullrich A and Schlessinger J (1990) Signal transduction by receptors with tyrosine kinase activity. *Cell* **61**: 203–212
Vossler MR, Yao H, York RD, Pan MG, Rim CS and Stork PJ (1997) cAMP activates MAP kinase and Elk-1 through a B-Raf- and Rap 1-dependent pathway. *Cell* **89**: 73–82
Withers DJ, Bloom SR and Rozengurt E (1995) Dissociation of cAMP-stimulated mitogenesis from activation of the mitogen-activated protein kinase cascade in Swiss 3T3 cells. *J Biol Chem* **270**: 21411–21419
Wu J, Dent P, Jelinek T, Wolfman A, Weber MJ and Sturgill TW (1993) Inhibition of the EGF-activated MAP kinase signaling pathway by adenosine 3′,5′-monophosphate. *Science* **262**: 1065–1069
Zuckier G and Tritton TR (1983) Adriamycin causes up regulation of epidermal growth factor receptors in actively growing cells. *Exp Cell Res* **148**: 155–161

© 1999 Cancer Research Campaign
